# High-frame-rate contrast-enhanced ultrasound particle image velocimetry in patients with a stented superficial femoral artery: a feasibility study

**DOI:** 10.1186/s41747-022-00278-w

**Published:** 2022-07-06

**Authors:** Majorie van Helvert, Stefan Engelhard, Jason Voorneveld, Marije van der Vee, Johan G. Bosch, Michel Versluis, Erik Groot Jebbink, Michel M. P. J. Reijnen

**Affiliations:** 1 Multi-Modality Medical Imaging Group, TechMed Centre, University of Twente, Enschede, The Netherlands; 2 Department of Vascular Surgery, Rijnstate Hospital, Arnhem, The Netherlands; 3 Physics of Fluids Group, TechMed Centre, University of Twente, Enschede, The Netherlands; 4 Department of Biomedical Engineering, Thorax Center, Erasmus Medical Center, Rotterdam, The Netherlands

**Keywords:** Microbubbles, Peripheral arterial disease, Rheology, Stents, Ultrasonography

## Abstract

**Background:**

Local blood flow affects vascular disease and outcomes of endovascular treatment, but quantifying it is challenging, especially inside stents. We assessed the feasibility of blood flow quantification in native and stented femoral arteries, using high-frame-rate (HFR) contrast-enhanced ultrasound (CEUS) particle image velocimetry (PIV), also known as echoPIV.

**Methods:**

Twenty-one patients with peripheral arterial disease, recently treated with a stent in the femoral artery, were included. HFR CEUS measurements were performed in the native femoral artery and at the inflow and outflow of the stent. Two-dimensional blood flow was quantified through PIV analysis. EchoPIV recordings were visually assessed by five observers and categorised as optimal, partial, or unfeasible. To evaluate image quality and tracking performance, contrast-to-tissue ratio (CTR) and vector correlation were calculated, respectively.

**Results:**

Fifty-eight locations were measured and blood flow quantification was established in 49 of them (84%). Results were optimal for 17/58 recordings (29%) and partial for 32 recordings (55%) due to loss of correlation (5/32; 16%), short vessel segment (8/32; 25%), loss of contrast (14/32; 44%), and/or shadows (18/32; 56%). In the remaining 9/58 measurements (16%) no meaningful flow information was visualised. Overall, CTR and vector correlation were lower during diastole. CTR and vector correlation were not different between stented and native vessel segments, except for a higher native CTR at the inflow during systole (*p* = 0.037).

**Conclusions:**

Blood flow quantification is feasible in untreated and stented femoral arteries using echoPIV. Limitations remain, however, none of them related to the presence of the stent.

**Trial registration:**

ClinicalTrials.gov, NCT04934501 (retrospectively registered).

**Supplementary Information:**

The online version contains supplementary material available at 10.1186/s41747-022-00278-w.

## Key points

- Ultrasound particle image velocimetry (echoPIV) enabled blood flow quantification inside the stented femoral artery.
- Multiple limiting issues of echoPIV were identified; however, none of them related to the stent.
- Several challenges must be overcome to bring echoPIV to clinical practice.

## Background

Atherosclerotic plaques typically localise at regions of the vascular tree with complex geometry, such as bifurcations and curvatures, where local disturbances in blood flow arise. Flow disturbances contribute to lower and irregular shear stresses which alter endothelial cell functioning with an overall proatherogenic environment as a result [1–3]. Local hemodynamics thus influence the onset and progression of vascular disease and outcomes of endovascular treatment. It is therefore of clinical significance to accurately visualise and quantify local blood flow patterns. However, real-time quantification of local hemodynamics in a fast and non-invasive way remains a great challenge.

In clinical practice, Doppler ultrasound (DUS) is commonly used to gain information on local hemodynamics to examine vascular pathology and outcomes of endovascular treatment of peripheral arterial disease. One important limitation of DUS remains that it solely provides the velocity component in the beam propagation direction with the assumption that the motion of blood is parallel to the vessel wall [4]. This assumption is often invalid in complex vessel geometries or near stenoses where complex flow patterns occur. As a result, the beam-to-flow angle is altered incorrectly causing inaccurate, highly operator-dependent, one-dimensional velocity estimations [5–8]. As a consequence, conventional DUS is unable to capture the full complexity of blood flow in a multi-dimensional manner and hence lacks information that may be of prognostic relevance.

In addition to DUS, computed tomography angiography is frequently used to assess vascular anatomy, stenosis grade, and stent patency. Without the addition of biomechanical flow modelling, this modality cannot be used to obtain functional information on hemodynamics.

Phase-contrast magnetic resonance imaging (PC MRI) overcomes the limitations of DUS and computed tomography angiography through allowing for blood flow characterisation in three dimensions [9]. However, PC MRI is time consuming, has a trade-off between spatial and temporal resolution, and has limited accessibility [9, 10]. Moreover, blood flow visualisation inside stents is compromised due to metal-related artefacts [11, 12]. These constraints inhibit the application of PC MRI for routine clinical use to evaluate local hemodynamics in and around stents.

Novel ultrasound (US) techniques have been developed to obtain probe angle-independent blood flow information. High-frame-rate (HFR) contrast-enhanced US (CEUS), in combination with particle image velocimetry (PIV), also known as echoPIV, is a technique enabling two-dimensional blood flow quantification. EchoPIV utilises microbubbles as an ultrasound contrast agent (UCA) to enhance the echogenicity of the blood pool to improve image quality [13]. Blood flow is characterised by tracking the displacement of a group of microbubbles on a frame-to-frame basis through cross-correlation analyses. Moreover, echoPIV employs unfocussed plane wave transmissions, allowing for HFR imaging (2,000−10,000 frames per second) and consequently tracking of high velocities and transient flow phenomena [14].

The two-dimensional velocity vector fields obtained with echoPIV can be used to visualise and quantify local blood flow patterns. This may give important new insight into (un)favourable flow patterns and flow-derived parameters in relation to atherosclerotic disease. Ultimately, these outcomes might predict disease progression and thereby possibly identify patients at risk of, for instance, in-stent restenosis prior to its occurrence. Thus far, echoPIV proved feasible for quantifying flow characteristics in the abdominal aorta of healthy volunteers [15], the left ventricle of patients with symptoms of heart failure [16], and the aortoiliac arteries of patients with aortoiliac occlusive disease [17].

Although these first *in vivo* results are promising, quantifying blood flow in stented vessels is likely more challenging due to the presence of stent material. Therefore, the aim of this study was to investigate the feasibility of imaging and quantification of blood flow patterns using echoPIV in peripheral arterial disease patients recently treated with a stent in the superficial femoral artery.

## Methods

### Study design and population

**Fig. 1:**
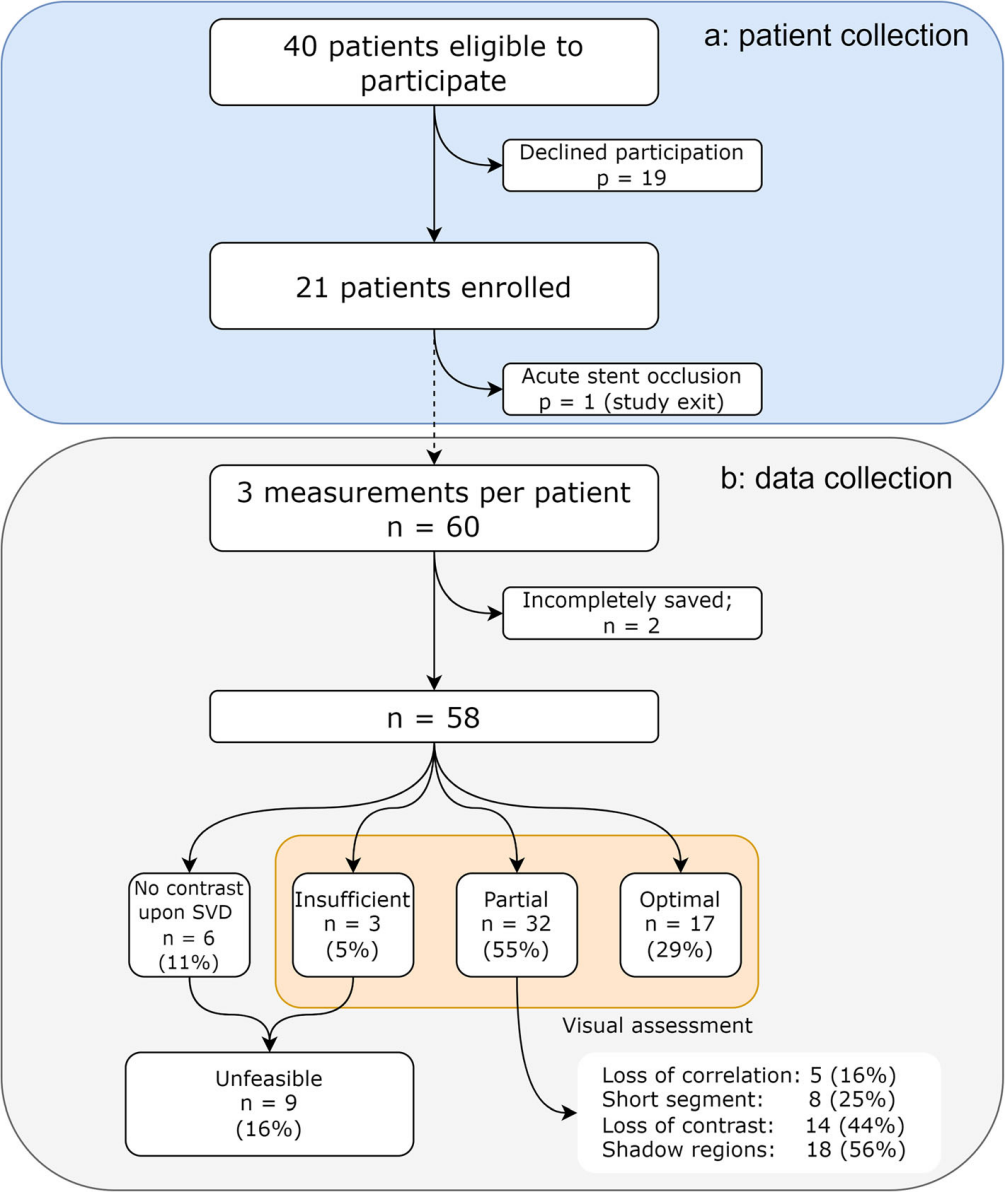
Enrolment (**a**) and data (**b**) flowchart of complete dataset considering the best measurement per location. *n* Number of measurements, *p* Number of patients, *SVD* Singular value decomposition

The study was approved by an authorised ethical committee in the Netherlands (NL65760.091.18) and the institutional review board, was conducted to conform to the Declaration of Helsinki, and was registered at ClinicalTrials.gov (NCT04934501). Patients with an atherosclerotic lesion of the superficial femoral artery and recently treated with a stent were prospectively enrolled in this single-institution exploratory trial. Patients with hypersensitivity to the excipients in the UCA or iodinated contrast, known history of severe cardiorespiratory diseases, uncontrolled systemic hypertension, hypercoagulable status, acute or recent (< 3 months) thrombosis, end-stage liver disease, glomerular filtration rate < 31 mL/min/1.73 m^2^, sepsis, or pregnancy were excluded. Procedures and treatment were executed per institutional protocol and standard of care. All patients provided written informed consent prior to enrolment in the study. An overview of the patient enrolment is presented in panel a of Fig. 1.

Clinical data on cardiovascular risk factors were collected and graded according to the Society for Vascular Surgery guidelines [18]. HFR CEUS measurements were performed within 6 to 8 weeks after treatment. Prior to the measurements, the femoral trajectory was examined using a clinical US scanner (iU22 xMATRIX, Philips Healthcare, Best, The Netherlands).

A computed tomography angiography was obtained as reference for vessel geometry and stent location (acquisition parameters provided in supplemental material, Appendix 1).

### Data acquisition

**Fig. 2:**
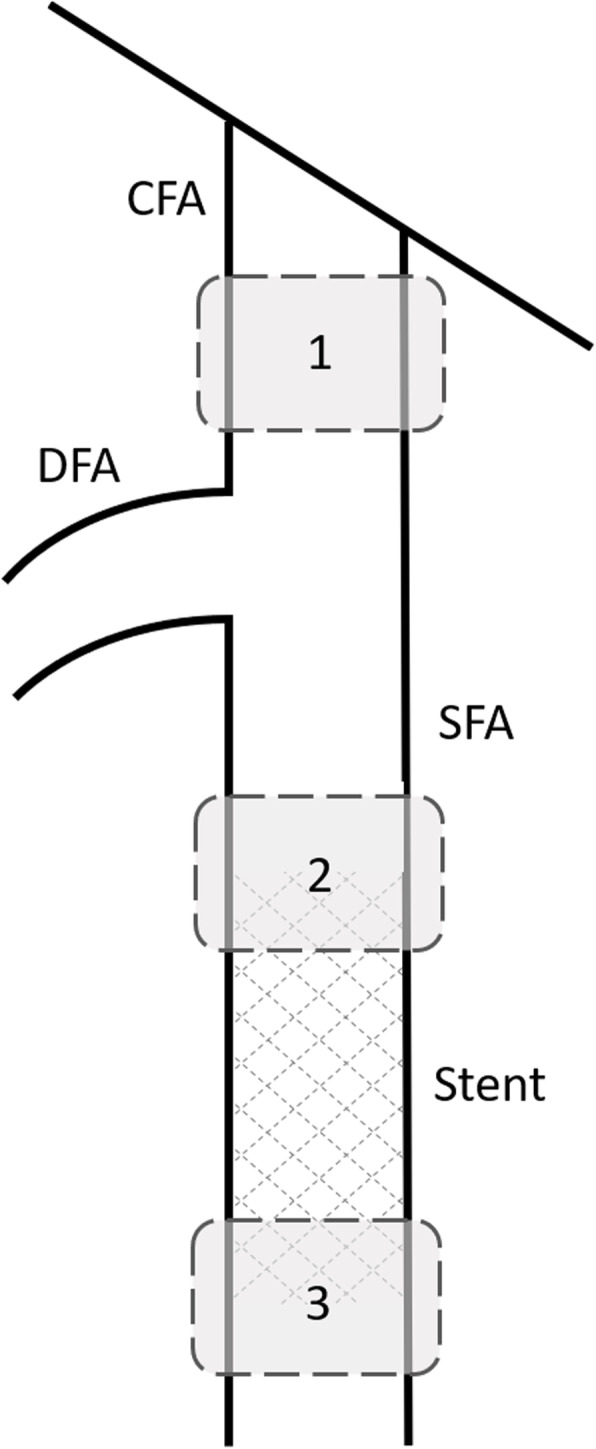
Schematic representation of the femoral bifurcation indicating the locations of interest. High-frame-rate contrast-enhanced radiofrequency data was obtained at the common femoral artery (1), inflow region of the stent (2), and the outflow region of the stent (3). *CFA* Common femoral artery, *DFA* Deep femoral artery, *SFA* Superficial femoral artery

HFR CEUS radiofrequency data were acquired with a fully programmable Vantage 256 US machine (Verasonics, Kirkland, WA) connected to a linear array transducer (Verasonics, Kirkland, WA; L11-4v; 6.25-MHz centre frequency). Measurements were performed at the common femoral artery (CFA), proximal edge (*i.e.*, inflow) and distal edge (*i.e.*, outflow) of the stent in the superficial femoral artery, as presented in Fig. 2. Prior to each measurement, 0.75 mL of UCA (Sonovue microbubbles; Bracco, Milan, Italy) was intravenously administered. Contrast levels were monitored using a pulse inversion scheme at a frame rate of 100 Hz [13]. After the injection bolus had passed and a semistable contrast concentration was observed, two HFR-CEUS measurements were performed consecutively with a transmit voltage of 5 and 10 V, corresponding to a mechanical index (MI) at the depth of interest (2−3 cm) of 0.06 and 0.12, respectively. Images were captured for 2.5 s using a 3-angled plane wave acquisition scheme with a pulse repetition frequency of 6 kHz. For each angle (−18°, 0°, and +18°), a 4-MHz single-cycle plane wave pulse was transmitted. To avoid accumulation of UCA, subsequent measurements were performed after complete washout of the contrast was observed.

### Image reconstruction

Data were processed offline using MATLAB R2019b (The Mathworks, Natick, MA, USA). Images were reconstructed using the Verasonics Vantage software (v4.0). All three angles were beamformed by means of delay-and-sum at the zero-degree beamforming grid, after which the beamformed in-phase quadrature data was coherently compounded. Consequently, a frame rate of 2 kHz was achieved.

A singular value decomposition (SVD) based clutter filter was applied to the compounded in-phase quadrature data to suppress tissue clutter [19]. The lower rank cut-off was automatically found based on the difference in spatial distribution between tissue and blood signal [20]. No higher rank cut-off was used to filter noise, as the bubble signal was still visible in the higher order singular values. When no contrast signal was visible after SVD filtering, the measurement was excluded for further PIV analysis and considered unfeasible.

### PIV analysis

PIV analysis was performed on the filtered in-phase quadrature data, after envelope detection, in an iterative manner with progressive grid refinement. Four iterations of blockwise normalised cross-correlation were calculated in the Fourier domain between each image pair, consisting of two iterations with a square block size of 6-mm interrogation windows and two iterations of 3 mm, all with 75% overlap. This corresponded to a vector grid spacing of 0.76 mm^2^. An ensemble correlation approach was used, averaging correlation maps of 10 consecutive frames. For each final correlation map, the location of the maximum was found to determine the displacement. Displacements based on a maximum correlation < 0.2 were disregarded, since these correlation values are close to the average noise level of the correlation map. Within each iteration, 2 × 3-pixel parabola peak fitting was used to estimate the sub-pixel displacement. Median outlier detection was applied to eliminate erroneous vectors. Removed vectors were replaced through linear interpolation. The obtained velocity data were smoothed using a temporal moving average filter (3 ensembles) and a spatial Gaussian filter (*σ* = 0.5 × 0.5; extent = 3 × 3).

### Qualitative assessment

Visual assessment of the velocity vectors was performed by five observers: (1) M.v.H., technical physician and PhD candidate with 3 years of experience in performing and reading echoPIV measurements counted from the introduction of the technique *in vivo*; (2) S.E., technical physician and PhD candidate, with 5 years of experience; (3) J.V., biomedical engineer and postdoctoral researcher, with 6 years of experience; (4) E.G.J., technical physician and assistant professor, with 6 years of experience; and (5) M.R., vascular surgeon and professor, with 6 years of experience. Methods for visual assessment were previously described by Engelhard et al. [17]. Briefly, each measurement was assigned to one of the following main categories: insufficient (no meaningful flow information could be derived hence considered to be unfeasible), partial (adequate blood flow quantification; however, one or multiple limiting issues occurred, as described in Table 1), or optimal (no limiting issues). Assessment took place individually. Final consensus between all five observers on the measurement with the best flow representation out of the two measurements per location and the corresponding feasibility score was reached during a consensus meeting.

**Table 1:** Issues limiting optimal flow quantification

Limiting issue	Description
Loss of correlation	Complete saturation of contrast image, high velocities/high spatial velocity gradients, or complex out-of-plane flow during systole causing a decreased cross-correlation in the PIV analysis due to loss of speckle coherency between frames. When a region of multiple vectors with cross-correlations < 0.2 existed, the “loss of correlation” category was selected.
Short vessel segment	Anatomical characteristics causing only a part of the vessel to be captured inside the imaged plane. When the length of the imaged vessel appeared smaller than 4 times its diameter, the “short vessel segment” category was selected.
Partial shadowing	Partial shadow regions with lower or no contrast signal, probably due to calcified lesions inside the image plane, compromising PIV analysis. When such a location with low contrast and subsequent spurious vectors appeared during the entire cardiac cycle, the “partial shadowing” category was selected.
Loss of contrast agent	Decrease in contrast signal during diastole due to severe destruction of the microbubbles because of the prolonged insonification period at lower velocities. When no vectors were obtained during multiple frames at the end of diastole, the “loss of contrast” category was selected.

*PIV* Particle image velocimetry, *UCA* Ultrasound contrast agent

### Quantitative assessment

**Fig. 3:**
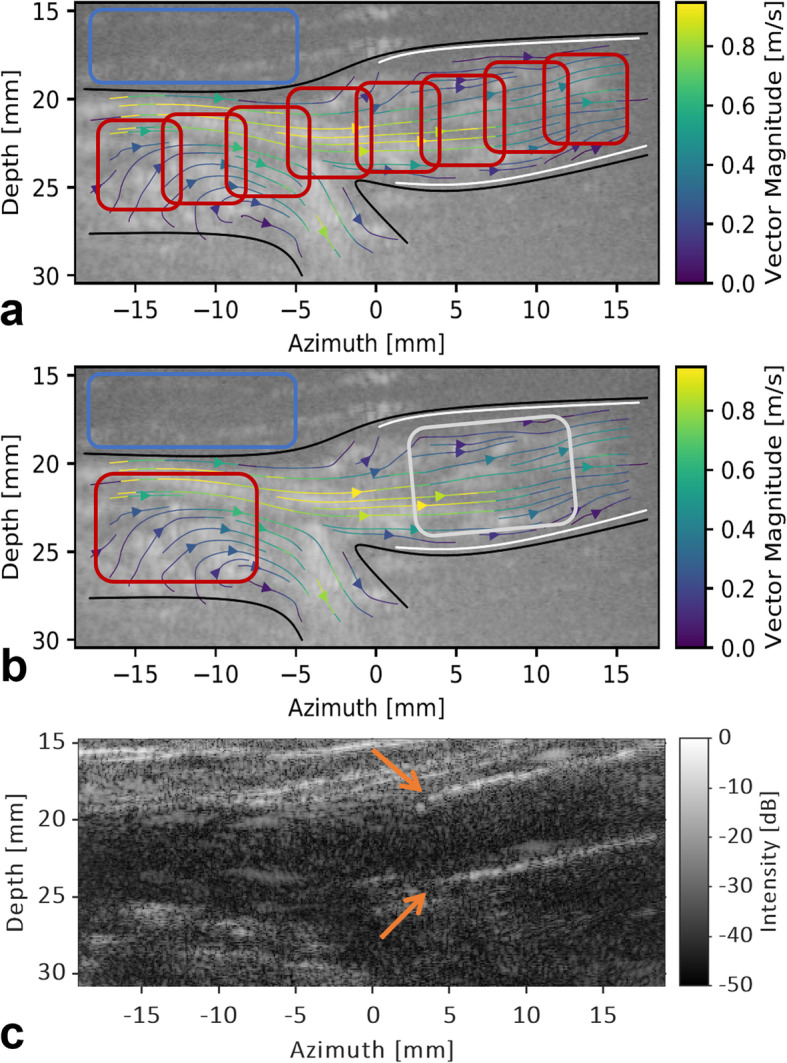
Example indicating the stent location and regions of interest (ROIs) used for the quantitative analysis. **a**, **b** Filtered contrast-enhanced US image captured at the proximal edge of the stent (white lines) with the velocity streamlines superimposed. **a** ROIs used to analyse the entire imaged vessel. To calculate the contrast-to-tissue ratio (CTR), the red squares were used for contrast signal strength whereas the blue square was used for tissue signal strength. Black lines represent the delineation of the imaged vessel. All vectors inside this (masked) region were used to calculate the average vector correlation. **b** ROIs used to analyse the stent influence. CTR was calculated for both the red (contrast strength in native vessel segment) and grey square (contrast strength in stented vessel segment) compared to the blue square. The average vector correlation was computed over all vectors inside the red and grey square for the native and stented segment, respectively. **c** B-mode image used to identify the stent transition. Orange arrows indicate the proximal edge of the stent

Signal strength of the administered contrast and tracking performance of PIV were quantified by calculating the contrast-to-tissue ratio (CTR) and normalised maximum vector correlation, respectively. Per frame, eight regions of interest along the imaged vessel, and one region of interest in the tissue above the imaged vessel, were selected (Fig. 3a). The CTR was then computed as $20\ {\mathit{\log}}_{10}\frac{RMS_{contrast}}{RMS_{tissue}}$, where *RMS* represents the root-mean-square signal strength of either the contrast or the tissue region. For each velocity vector field, an average PIV, *i.e.*, vector, correlation value, ranging between 0 and 1, was calculated over all vectors. Both measures were obtained during the systolic and diastolic phase of the cardiac cycle. Systole and diastole were automatically identified on the basis of the inflexion points of the acquired temporal velocity profile at the centreline of the imaged vessel.

### Feasibility of echoPIV in stented vessels

Data of the inflow and outflow locations of the stent were used to evaluate the influence of the stent material on the CTR and vector correlation. Both measures were calculated for a region of interest selected in a stented segment and native segment of the imaged vessel (Fig. 3b, c). Only measurements categorised as optimal, and in which both a stented and native vessel segment could be clearly identified, were considered. Data obtained with an MI of 0.06 and 0.12 were pooled.

### Statistical analysis

Statistical analysis was performed using IBM SPSS statistics 26 (IBM corporation, Armonk, NY, USA). Categorical variables are expressed as number (%) and continuous variables are given as median (interquartile range). Interobserver agreement between the five observers on the three main categories was investigated by calculating the interclass correlation coefficient based on a mean-rating (*k* = 5), absolute-agreement, two-way mixed-effects model [21]. Differences in CTR and vector correlation between systole and diastole, and between an MI of 0.06 and 0.12, were tested with a Mann-Whitney *U* test for non-parametric data, given the small sample sizes. To evaluate the influence of bubble destruction on these metrics, the same tests were performed after exclusion of the cases assigned to the loss of contrast agent feasibility category by the observers. *p*-values < 0.05 were considered statistically significant.

## Results

Twenty-one consecutive patients were included in the study. One patient was excluded as the clinical US device revealed a stent thrombosis. Baseline characteristics of the remaining cohort are presented in Table 2, with reference to the Society for Vascular Surgery [18] and the TransAtlantic Inter-Society Consensus II [22]. A bare nitinol stent (Everflex; Medtronic, Minneapolis, MN, USA) was used in 55% (*n* = 11), while a covered nitinol stent (Viabahn; W.L. Gore and Associates, Flagstaff, AZ, USA) was placed in 45% of patients (*n* = 9). On average measurements were performed 40 days (31–50 days) after treatment. Our protocol dictated three measurement locations per patient and considering only the best recording out of two applied MIs, the dataset should consist of 3 times 20 recordings. However, incomplete saving due to a disc full error caused two measurements to be lost, resulting in a final dataset of 58 echoPIV recordings. An overview of the data collection is presented in panel b of Fig. 1.

**Table 2:** Patient demographics and lesion characteristics (*n* = 20)

Age, years	71 (66–78)
Gender	13 males (65%); 7 females (35%)
Cardiovascular risk factors (Society for Vascular Surgery grade (0; 1; 2; or 3) ^a^)	
Diabetes mellitus	13 (65%); 1 (5%); 5 (25%); 1 (5%)
Tobacco use	15 (75%); 4 (20%); 0 (0%); 1 (5%)
Hypertension	4 (20%); 6 (30%); 5 (25%); 5 (25%)
Renal insufficiency	19 (95%); 1 (5%); (0%); (0%)
Hyperlipidemia	6 (30%); (0%); (0%); 14 (70%)
Cardiac disease	17 (85%); 1 (5%); 2 (10%); (0%)
Pulmonary disease	16 (80%); 4 (20%); (0%); (0%)
TransAtlantic Inter-Society Consensus II class (A; B; C; or D) ^b^	6 (30%); 5 (25%); 5 (25%); 4 (20%)

Values are presented as median (interquartile range) or number and percentage

^a^Reference no. [18], ^b^ reference no. [22]

### Qualitative assessment

**Fig. 4:**
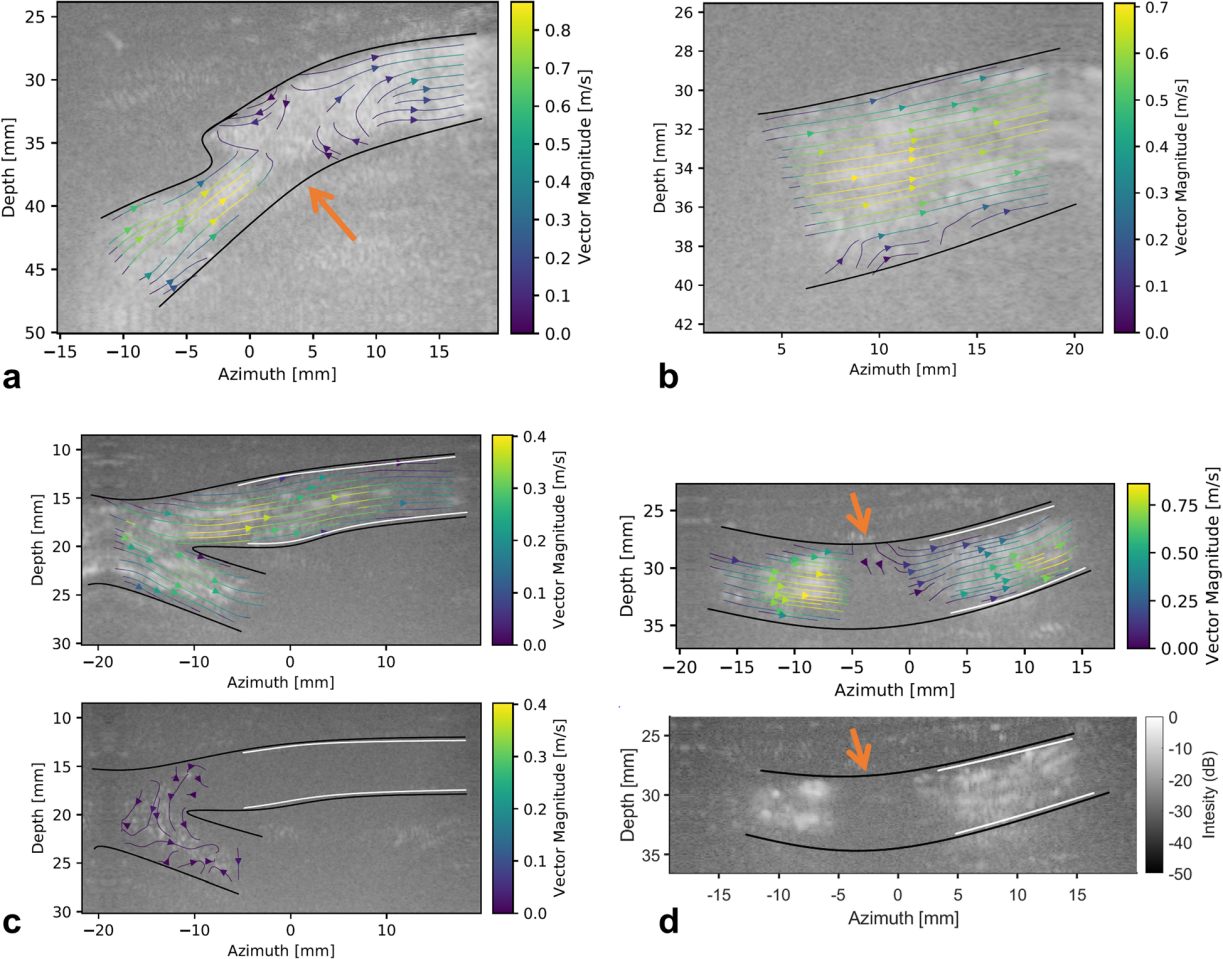
Example of each limiting issue causing partial flow quantification. Filtered contrast-enhanced US images with velocity streamlines superimposed. The vessel wall is presented in black in all examples. The presence of a stent is indicated in white. **a** Loss of correlation due to high velocities or complex flow phenomena, here observed distal to a stenotic lesion, indicated with the orange arrow (supplementary video 4). **b** Only a small part of the vessel is captured within the field of view due to the geometry of the arteries (supplementary video 5). **c** Loss of contrast due to microbubble destruction. During systole (top), constant replenishment of microbubbles permits sufficient bubble signal, whereas severe bubble destruction occurs during diastole (bottom) due to the prolonged insonation period in the case of lower blood flow velocities (supplementary video 6). **d** A shadow region possibly caused by a calcified lesion at the anterior side of the lumen, resulting in an interruption of the vector velocity field (top) and bubble intensity (bottom) (supplementary video 7)

Blood flow quantification was feasible in 49 out of 58 (84%) measurements, of which 17 were considered to be optimal (29%; supplementary videos 1, 2, and 3). Partial flow quantification was observed in 32 out of 58 (55%) measurements due to loss of correlation during systole (5/32 cases, 16%; Fig. 4a and supplementary video 4), a short vessel segment imaged (8/32 cases, 25%; Fig. 4b and supplementary video 5), loss of contrast agent during diastole (14/32 cases, 44%; Fig. 4c and supplementary video 6), and the presence of a shadow (18/32 cases, 56%; Fig. 4d and supplementary video 7). Nine out of 58 (16%) measurements were considered unfeasible since no meaningful information could be derived from the data due to lack of contrast after SVD filtering (6/9 cases; 67%) or insufficient flow quantification based on visual assessment (3/9 cases; 33%). The interobserver agreement was 0.647 (95% confidence interval 0.645–0.649). An overview of the feasibility assessment per location and the occurrence of each limiting issue is provided in Tables 3 and 4, respectively.

**Table 3:** Feasibility assessment per location

Location	Feasibility category			
	No contrast upon SVD, *n* (%)	Insufficient, *n* (%)	Partial, *n* (%)	Optimal, *n* (%)
CFA (*n* = 20)	2 (10%)	2 (10%)	8 (40%)	8 (40%)
Inflow (*n* = 19)	1 (5%)	0 (0%)	14 (74%)	4 (21%)
Outflow (*n* = 19)	3 (16%)	1 (5%)	10 (53%)	5 (26%)
Total (*n* = 58)	6 (11%)	3 (5%)	32 (55%)	17 (29%)

*CFA* Common femoral artery*, SVD* Singular value decomposition

**Table 4:** Partial feasibility: limiting issue per location

Location	Limiting issues			
	Loss of correlation, *n* (%)	Short segment, *n* (%)	Loss of contrast agent, *n* (%)	Shadow regions, *n* (%)
CFA (*n* = 8)	3 (38%)	3 (38%)	2 (25%)	4 (50%)
Inflow (*n* = 14)	1 (7%)	2 (14%)	7 (50%)	8 (57%)
Outflow (*n* = 10)	1 (10%)	3 (30%)	5 (50%)	6 (60%)
Total (*n* = 32)	5 (16%)	8 (25%)	14 (44%)	18 (56%)

*CFA* Common femoral artery

### Quantitative assessment

**Fig. 5:**
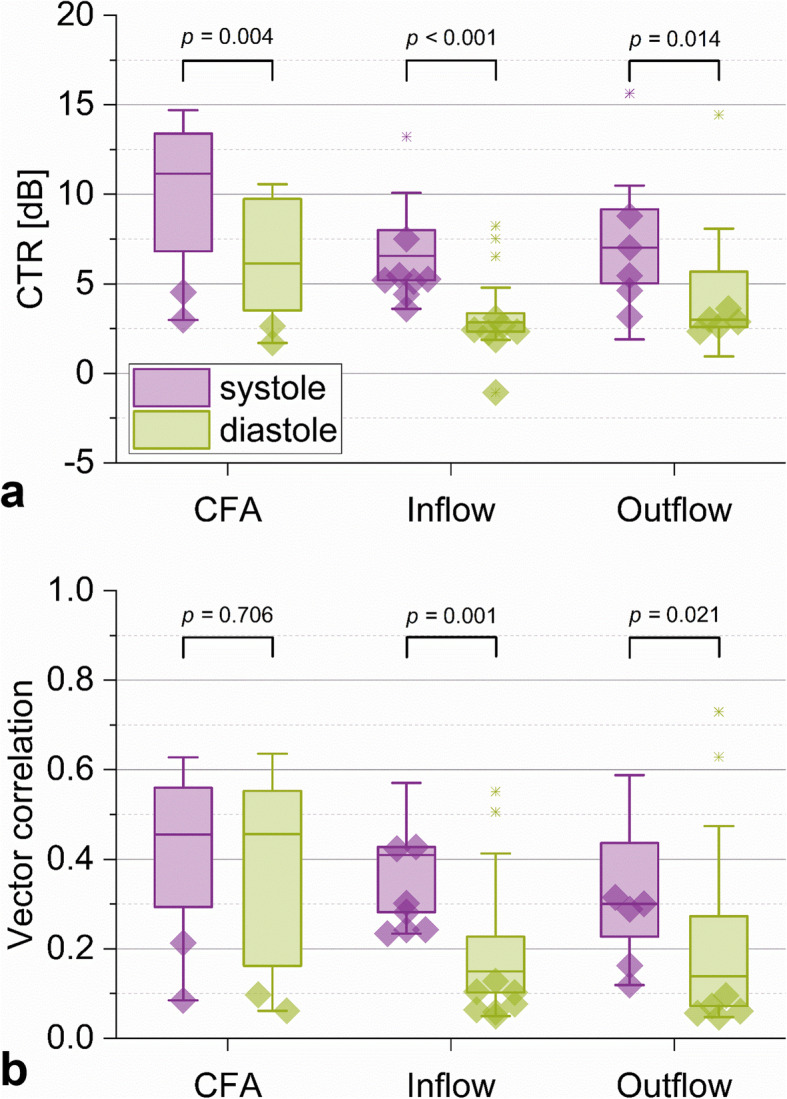
Comparison of contrast-to-tissue ratio (CTR) (**a**) and vector correlation (**b**) between systole (purple) and diastole (green) per location. Edges of the boxes indicate the 25th (Q1) and 75th (Q3) percentiles, whereas whiskers give the minimum and maximum values. Outliers are presented as crosses. The values corresponding to measurements assigned as “loss of contrast” by the observers are indicated with a diamond shape.*p < 0.05, *ns* Not significant, *CFA* Common femoral artery

Figure 5 shows the CTR and vector correlation during systole and diastole for all three locations. CTR decreased significantly during diastole at all three locations (CFA; *p* = 0.004, inflow; *p* < 0.001 and outflow; *p* = 0.014). Vector correlation decreased significantly during diastole as well (inflow; *p* = 0.001 and outflow; *p* = 0.021), except at the CFA (*p* = 0.706).

After excluding the cases with severe loss of contrast agent during diastole (*n* = 14, Table 4) from the statistical tests, CTR and vector correlation still reduced significantly during diastole at the inflow (*p* = 0.003 and *p* = 0.033, respectively). At the outflow, conversely, no significant differences in CTR and vector correlation between systole and diastole were found (*p* = 0.112 and *p* = 0.226, respectively).

Detailed results of the evaluation of all measurements are given in supplemental Tables S1 and S2. Quantitative results for both MIs are provided in supplementary Fig. S1. No significant differences were found in CTR and vector correlation between the measurements obtained with an MI of either 0.06 or 0.12.

### Feasibility of echoPIV in a stent

**Fig. 6:**
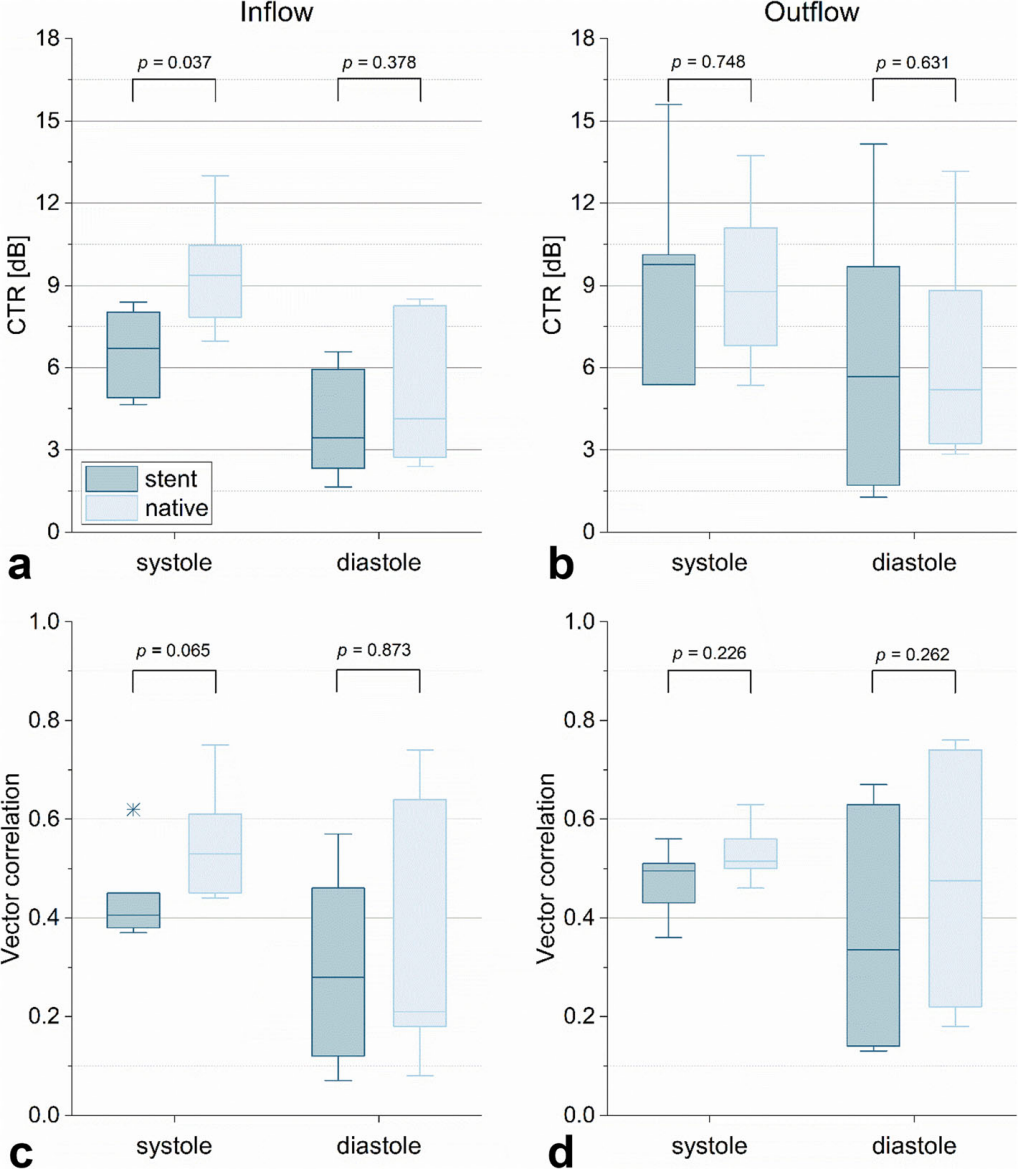
Comparison of contrast-to-tissue ratio (CTR) (**a**, **b**) and vector correlation (**c**, **d**) between a stented and native vessel segment. Stented segment is presented in dark blue, whereas native segment is given in light blue. Edges of the boxes indicate the 25th (Q1) and 75th (Q3) percentiles, whereas whiskers give the minimum and maximum values. Outliers are presented as crosses. *p < 0.05, *ns* Not significant

Six measurements (Everflex; *n* = 5, Viabahn; *n* = 1) at both the inflow (supplementary videos 8 and 9) and outflow (supplementary videos 10 and 11) of the stent were eligible for further analysis to quantify the influence of the stent. An overview of the results is given in Fig. 6. No differences were found in CTR and vector correlation between a native and stented segment for the inflow and outflow of the stent, except a significantly higher systolic CTR for the native segment at the inflow: 9.4 (7.8–10.5) *versus* 6.7 (4.9–8.0), *p* = 0.037.

## Discussion

The present work investigated whether echoPIV is feasible near and inside stents as a first step towards the perspective of complex vascular blood flow quantification. Outcomes show that echoPIV is able to quantify two-dimensional blood flow patterns in the native and stented femoral artery. The stent material did not hinder the measurements, indicating the robustness of the US contrast technique to stenting.

Visual assessment of the echoPIV recordings revealed optimal flow quantification in 29% of the measurements, while partial flow quantification was established in 55% of the measurements. In the latter case, blood flow could still be quantified during a part of the cardiac cycle or in a part of the vessel. Several limiting issues were defined and highlight the difficulty of blood flow quantification in peripheral arterial disease patients. Some of these issues impede US examinations in general, whereas others are specifically related to echoPIV.

Optimal blood flow quantification was mostly hindered by shadow regions due to calcified lesions in the imaged plane. Shadows observed inside the stent were most likely caused by the primary, treated, lesion. The high scattering properties of a calcification prevent the transmitted US signal to reach the blood pool. This remains an unsolved problem for all US-based blood flow imaging techniques and would demand complex US transmit and receive sequences to compensate for the scattering effect of the calcifications.

In addition, multiple cases showed impaired blood flow quantification during diastole due to loss of contrast agents. This issue is more specifically related to the stability of UCA upon multiple US insonations. The prolonged period of insonation at lower velocities increases the severity of bubble destruction [23, 24], which corresponds to the lower CTR during diastole found in this study. Cases with severe bubble destruction showed correlation values close to the noise floor of the correlation map, and as a result, no meaningful blood flow information could be obtained. Fortunately, “moderate” bubble destruction did not affect the tracking of blood flow. Destruction of UCA is directly related to the transmitted US pressures. Voorneveld et al. [24] described “severe” destruction at an MI of 0.06 or higher [25]. Thus, the MI of 0.06 and 0.12 used in the current study are relatively high. To reduce UCA destruction, lower MIs could be applied, but at the cost of signal-to-noise ratio. Optimal flow quantification was mostly observed in cases acquired with an MI of 0.12, indicating that whenever bubble destruction was limited, the increased signal-to-noise ratio indeed provides better results. A novel class of UCA, either more stable or with improved scattering characteristics, may overcome these limitations [26]. Currently, only polydisperse microbubble suspensions, including Sonovue, are commercially available for clinical use. Given the broad size distribution of the microbubbles (between 1 and 10 μm) and the inverse relation between microbubble size and resonance frequency, only a small fraction of the microbubbles will exhibit resonance behaviour at any given US frequency. Scattering characteristics can be improved by using a suspension of monodisperse microbubbles [26]. *In vitro* experiments already showed a two-fold increase in sensitivity of monodisperse microbubbles compared to a polydisperse suspension. Moreover, the resonance behaviour of such a monodisperse population appears more non-linear [27]. Dedicated non-linear pulsing schemes can utilise these increased non-linear properties to better distinguish between the microbubbles and the surrounding tissue clutter [13].

Finally, several recordings showed only a small segment of the targeted vessel or reduced blood flow quantification during systole due to loss of correlation. The former issue is intrinsic to the tortuous anatomy of blood vessels. 4D echoPIV could resolve this matter and would allow for the quantification of complex, out-of-plane, flow phenomena. Development of this technique for *in vivo* use is still in a preliminary stage [28, 29]. The latter issue was predominantly observed near stenotic lesions, indicating it originated from high velocities (> 120 cm/s)—with high spatial gradients—or complex, out-of-plane flow that cannot be accurately tracked by current PIV analysis. Correlation compounding was performed to refine tracking performance in the presence of high velocities, as suggested by Leow et al. [30], but no improvements were observed. Moreover, in other recordings, decorrelation appeared to be related to high UCA concentrations causing complete saturation of the image. As a consequence, the inter-frame decorrelation of the resulting speckle pattern increased which limited PIV tracking capabilities. The analysis could have benefitted from lowering the amount of injected UCA allowing for images with a sparser speckle [31, 32].

Fortunately, none of the previously discussed issues relate to the stent material. Also, no significant differences were found in CTR and vector correlation between native and stented vessel segments, except for the systolic CTR at the inflow region of the stent. This higher CTR of the native segment at the inflow may be explained by the presence of the stent [33]. Furthermore, due to longer exposure to US, UCA destruction increases while moving downstream along the stented vessel (from left to right) [25]. Nevertheless, the vector correlation did not show a significant decrease in the stented segment. At the outflow region, where the stent is located upstream, no difference was found in CTR between the native and stented segments, suggesting that both effects, *i.e.*, the stent material and bubble destruction, compensate each other.

All data processing was performed off-line. Overall feasibility outcomes could improve when immediate feedback is provided during the measurements to allow for adjustments in US settings and desired imaging plane per individual patient. This would be a key improvement for the technique to be of added value in clinical practice.

The velocity vector field obtained with echoPIV can be used to visualise and quantify two-dimensional blood flow patterns and subsequently derive blood flow parameters, such as vector complexity [34, 35] and vorticity [36]. To further investigate blood flow patterns and flow-derived parameters in relation to vascular disease progression, longitudinal clinical trials with larger patient cohorts are required. Such a clinical trial is essential to illustrate the clinical implications and prognostic value echoPIV may have in identifying (un)favourable hemodynamic conditions and, for instance, patients at risk of in-stent restenosis. Ultimately, this modality could provide an important step to improve patient-specific therapy and aid in durable treatment and a cost-effective follow-up, based on individual risks.

The present study has several limitations that should be considered. First, the study is limited by its small sample size, especially for the assessment of the stent influence on echoPIV results. Second, feasibility of echoPIV was not validated by comparing the outcomes with a different technique. Although velocities were obtained with DUS, no valid comparison could be made mainly because of inaccurate angle-correction and difficulties in flow direction estimates. PC MRI scans were not acquired because of the expected stent-related artefacts [11, 12]. Nevertheless, visual evaluation of the data revealed a close match between the movement of the UCA and the superimposed velocity vectors and thus the echoPIV results were assumed to be valid. Also, the inter-observer agreement on the three main feasibility categories was moderate. Though considered sufficient given the different training backgrounds of the five observers, it emphasises the subjectivity of the assessment and the complexity of the data. Nonetheless, results of the visual assessment do comply with the results of the quantitative measures, as recordings in which severe bubble destruction was observed corresponded to lower CTR and vector correlation values, suggesting that the visual evaluation is a justified method to assess the feasibility of the echoPIV technique.

In conclusion, this study shows that echoPIV is able to quantify blood flow in both the native and stented femoral artery, providing an important first step towards the perspective of complex vascular blood flow quantification. Using echoPIV, angle-independent velocities, in the form of a two-dimensional vector field, can be obtained which allows for the visualisation of short-lived and complex blood flow patterns. Several mechanisms that hinder echoPIV from optimal two-dimensional blood flow quantification, including bubble destruction and out-of-plane flow, were identified. For the technique to be of added value in clinical practice, further developments such as three-dimensional acquisitions and real-time feedback should be investigated. Fortunately, optimal blood flow quantification was not impeded by the stent material, indicating the robustness of echoPIV in the presence of a stent.

## Supplementary Information

**Additional file 1: Table S1.** Feasibility assessment per location for both MIs and the best measurement. **Table S2.** Partial feasibility: limiting issue per location for both MIs and the best measurement. **Figure S1.** Comparison of contrast-to-tissue ratio (CTR) (a, b, c) and vector correlation (d, e, f) between systole (purple) and diastole (green) per location for both mechanical indexes (MI). Edges of the boxes indicate the 25th (Q1) and 75th (Q3) percentiles, whereas whiskers give the minimum and maximum values. Outliers are presented as crosses. The values corresponding to measurements assigned as “loss of contrast” by the observers are highlighted with a diamond shape.* *p* < 0.05, ns Not significant, 0.06 Measurements with an MI of 0.06, 0.12 Measurements with an MI of 0.12.

**Additional file 2: Video 1.** EchoPIV recording (2.5 s, slowed down to 16 s) of the blood flow in the common femoral artery. Vector velocity data of about 3 heart cycles is presented, as indicated by the temporal velocity profile. Distal to the stenosis (azimuth = -2 mm) recirculating blood flow is visualised. The colour and size of the vectors represent the flow velocities. Vectors based on a correlation < 0.2 are given as red dots and considered to be erroneous.

**Additional file 3: Video 2.** EchoPIV recording (2.5 s, slowed down to 16 s) of the blood flow in the common femoral artery. Vector velocity data of about 5 heart cycles is presented, as indicated by the temporal velocity profile. This patient presented with a cardiac arrhythmia. The colour and size of the vectors represent the flow velocities. Vectors based on a correlation < 0.2 are given as red dots and considered to be erroneous.

**Additional file 4: Video 3.** EchoPIV recording (2.5 s, slowed down to 16 s) of the blood flow in the common femoral artery. Vector velocity data of about 3 heart cycles is presented, as indicated by the temporal velocity profile. Velocities up to 120 cm/s are captured. The colour and size of the vectors represent the flow velocities. Vectors based on a correlation < 0.2 are given as red dots and considered to be erroneous.

**Additional file 5: Video 4.** EchoPIV recording (2.5 s, slowed down to 16 s) of the blood flow in the common femoral artery. Vector velocity data of about 2 heart cycles is presented, as indicated by the temporal velocity profile. High and disturbed (out-of-plane) blood flow velocities, caused by a stenotic lesion (azimuth = -1), cannot be adequately captured (Fig. 4a). The colour and size of the vectors represent the flow velocities. Vectors based on a correlation < 0.2 are given as red dots and considered to be erroneous.

**Additional file 6: Video 5.** EchoPIV recording (2.5 s, slowed down to 16 s) of the blood flow at the inflow of the stent in the superficial femoral artery. Vector velocity data of about 2 heart cycles is presented, as indicated by the temporal velocity profile. During diastole the microbubbles are destroyed due to the prolonged exposure time to ultrasound. Consequently, blood flow could not be visualised during this phase of the cardiac cycle (Fig. 4d). Vectors based on a correlation < 0.2 are given as red dots and considered to be erroneous.

**Additional file 7: Video 6.** EchoPIV recording (2.5 seconds, slowed down to 16 seconds) of the blood flow at the inflow of the stent in the superficial femoral artery. Vector velocity data of about 3 heart cycles is presented, as indicated by the temporal velocity profile. Only a short segment of the vessel (without the stent) is captured in the image plane (Fig. 4b). The colour and size of the vectors represent the flow velocities. Vectors based on a correlation < 0.2 are given as red dots and considered to be erroneous.

**Additional file 8: Video 7.** EchoPIV recording (2.5 s, slowed down to 16 s) of the blood flow at the inflow of the stent in the superficial femoral artery. Vector velocity data of about 3 heart cycles is presented, as indicated by the temporal velocity profile. A calcification (azimuth = from -5 to 0 mm) causes a shadow region at which the blood flow cannot be quantified throughout the entire cardiac cycle (Fig. 4c). The colour and size of the vectors represent the flow velocities. Vectors based on a correlation < 0.2 are given as red dots and considered to be erroneous.

**Additional file 9: Video 8.** B-mode contrast recording at the inflow region of the stent in the superficial femoral artery used to monitor contrast levels and as reference for the stent location. The proximal edge of the stent can be found around 5 mm azimuth.

**Additional file 10: Video 9.** EchoPIV recording (2.5 s, slowed down to 16 s) of the blood flow at the inflow of the stent in the superficial femoral artery. Vector velocity data of about 2 heart cycles is presented, as indicated by the temporal velocity profile. The stent was placed in the proximal superficial femoral artery and thus the femoral bifurcation is imaged. The colour and size of the vectors represent the flow velocities. Vectors based on a correlation < 0.2 are given as red dots and considered to be erroneous.

**Additional file 11: Video 10.** B-mode contrast recording at the outflow region of the stent in the superficial femoral artery used to monitor contrast levels and as reference for the stent location. The distal edge of the stent can be found around -5 mm azimuth.

**Additional file 12: Video 11.** EchoPIV recording (2.5 s, slowed down to 16 s) of the blood flow at the outflow of the stent in the superficial femoral artery. Vector velocity data of about 3 heart cycles is presented, as indicated by the temporal velocity profile. Venous blood flow can be appreciated posterior to the superficial femoral artery. The colour and size of the vectors represent the flow velocities. Vectors based on a correlation < 0.2 are given as red dots and considered to be erroneous.

## Data Availability

The datasets generated and/or analysed during the current study are not publicly available because it consists in part of personal (pseudo-anonymised) patient data. The processed data are available from the corresponding author on reasonable request.

## Abbreviations

CEUS: Contrast-enhanced ultrasound
CFA: Common femoral artery
CTR: Contrast-to-tissue ratio
DUS: Doppler ultrasound
HFR: High-frame-rate
MI: Mechanical index
PC MRI: Phase-contrast magnetic resonance imaging
PIV: Particle image velocimetry
SVD: Singular value decomposition
UCA: Ultrasound contrast agent
US: Ultrasound
