# Supplementary material for: High-frame-rate contrast-enhanced ultrasound particle image velocimetry in patients with a stented superficial femoral artery: a feasibility study
Source: Eur Radiol Exp. 2022 Jul 6;6:32. doi: 10.1186/s41747-022-00278-w (PMC9256892; doi:10.1186/s41747-022-00278-w)
Supplement: Supplementary file 1 — Additional file 1: Table S1. Feasibility assessment per location for both MIs and the best measurement. Table S2. Partial feasibility: limiting issue per location for both MIs and the best measurement. Figure S1. Comparison of contrast-to-tissue ratio (CTR) (a, b, c) and vector correlation (d, e, f) between systole (purple) and diastole (green) per location for both mechanical indexes (MI). Edges of the boxes indicate the 25th (Q1) and 75th (Q3) percentiles, whereas whiskers give the minimum and maximum values. Outliers are presented as crosses. The values corresponding to measurements assigned as “loss of contrast” by the observers are highlighted with a diamond shape.* p < 0.05, ns Not significant, 0.06 Measurements with an MI of 0.06, 0.12 Measurements with an MI of 0.12. [file 41747_2022_278_MOESM1_ESM.docx]

**ELECTRONIC SUPPLEMENTARY MATERIAL**

**High-frame-rate contrast-enhanced ultrasound particle image velocimetry in patients with a stented superficial femoral artery: a feasibility study**

**Appendix 1** – acquisition parameters computed tomography angiography scan

Computed tomography angiography scans were acquired using a 256-slice computed tomography scanner (Brilliance iCT 256, Philips Healthcare, Best, The Netherlands) with a standardized arterial scan protocol. The scan parameters were as follows: tube voltage, 100 or 120 kV; tube current time product, median 108.5 mA ∙ s (range: 60 – 249); pitch factor, 0.34; collimation, 80 × 0.625 mm; slice thickness, 0.9 mm; slice increment, 0.45 mm; reconstruction matrix, 512 × 512 pixels; dose length product, median 494.5 mGy ∙ cm (range: 209.4 – 1330). In total a maximum of 110 mL radiocontrast agent (Xenetix 350) was intravenously administered at a rate of 4 mL/s (range: 2 – 4.5, depending on patient’s body weight).

**Table S1.** Feasibility assessment per location for both MIs and the best measurement.

| **Location** | **Feasibility category** | | | | | | | | | | | |
| --- | --- | --- | --- | --- | --- | --- | --- | --- | --- | --- | --- | --- |
|  | **No contrast upon SVD, n(%)** | | | **Insufficient, n(%)** | | | **Partial, n(%)** | | | **Optimal, n(%)** | | |
|  | **0.06** | **0.12** | **Best** | **0.06** | **0.12** | **Best** | **0.06** | **0.12** | **Best** | **0.06** | **0.12** | **Best** |
| **CFA** (n_0.06_ = 20, n_0.12_ = 19) | 3  (15%) | 2  (11%) | 2  (10%) | 5  (25%) | 1  (5%) | 2  (10%) | 8  (40%) | 9  (47%) | 8  (40%) | 4  (20%) | 7  (37%) | 8  (40%) |
| **Inflow** (n_0.06_ = 19, n_0.12_ = 18) | 1  (5%) | 1  (6%) | 1  (5%) | 1  (5%) | 0  (0%) | 0  (0%) | 15 (79%) | 13  (72%) | 14  (74%) | 2  (11%) | 4  (22%) | 4  (21%) |
| **Outflow** (n_0.06_ = 19, n_0.12_ = 19) | 4  (21%) | 3  (16%) | 3  (16%) | 3  (16%) | 2  (11%) | 1  (5%) | 11  (58%) | 9  (47%) | 10  (53%) | 1  (5%) | 5  (26%) | 5  (26%) |
| **Total** (n_0.06_ = 58, n_0.12_ = 56) | 8  (14%) | 6  (11%) | 6  (11%) | 9  (15%) | 3  (5%) | 3  (5%) | 34  (59%) | 31  (55%) | 32  (55%) | 7  (12%) | 16 (29%) | 17  (29%) |

CFA; common femoral artery, n_0.06_; number of measurements with an MI of 0.06, n_0.12_; number of measurements with an MI of 0.12.

**Table S2.** Partial feasibility: limiting issue per location for both MIs and the best measurement.

| **Location** | **Limiting issues** | | | | | | | | | | | |
| --- | --- | --- | --- | --- | --- | --- | --- | --- | --- | --- | --- | --- |
|  | **Loss of correlation, n(%)** | | | **Short segment, n(%)** | | | **Loss of contrast agent, n(%)** | | | **Shadow regions, n(%)** | | |
|  | **0.06** | **0.12** | **Best** | **0.06** | **0.12** | **Best** | **0.06** | **0.12** | **Best** | **0.06** | **0.12** | **Best** |
| **CFA** (n_0.06_ = 8, n_0.12_ = 9) | 3  (38%) | 4  (44%) | 3  (38%) | 1  (13%) | 3  (33%) | 3  (38%) | 0  (0%) | 2  (22%) | 2  (25%) | 4  (50%) | 4  (44%) | 4  (50%) |
| **Inflow** (n_0.06_ = 15, n_0.12_ = 13) | 1  (7%) | 1  (8%) | 1  (7%) | 2  (13%) | 2  (15%) | 2  (14%) | 7  (47%) | 7  (54%) | 7  (50%) | 9  (60%) | 8  (62%) | 8  (57%) |
| **Outflow** (n_0.06_ = 11, n_0.12_ = 9) | 1  (9%) | 1  (11%) | 1  (10%) | 5  (45%) | 2  (22%) | 3  (30%) | 4  (36%) | 5  (56%) | 5  (50%) | 7  (64%) | 5  (56%) | 6  (60%) |
| **Total** (n_0.06_ = 34, n_0.12_ = 31) | 5  (18%) | 6  (19%) | 5  (16%) | 8  (24%) | 7  (23%) | 8  (25%) | 11  (32%) | 14  (45%) | 14  (44%) | 20  (59%) | 17  (55%) | 18  (56%) |

CFA; common femoral artery, n_0.06_; number of measurements with an MI of 0.06, n_0.12_; number of measurements with an MI of 0.12.

**Figure S1.** Comparison of contrast-to-tissue ratio (CTR) (**a**, **b**, **c**) and vector correlation (**d**, **e**, **f**) between systole (purple) and diastole (green) per location for both mechanical indexes (MI). Edges of the boxes indicate the 25th (Q1) and 75th (Q3) percentiles, whereas whiskers give the minimum and maximum values. Outliers are presented as crosses. The values corresponding to measurements assigned as “loss of contrast” by the observers are highlighted with a diamond shape.* p < 0.05, ns Not significant, 0.06 Measurements with an MI of 0.06, 0.12 Measurements with an MI of 0.12


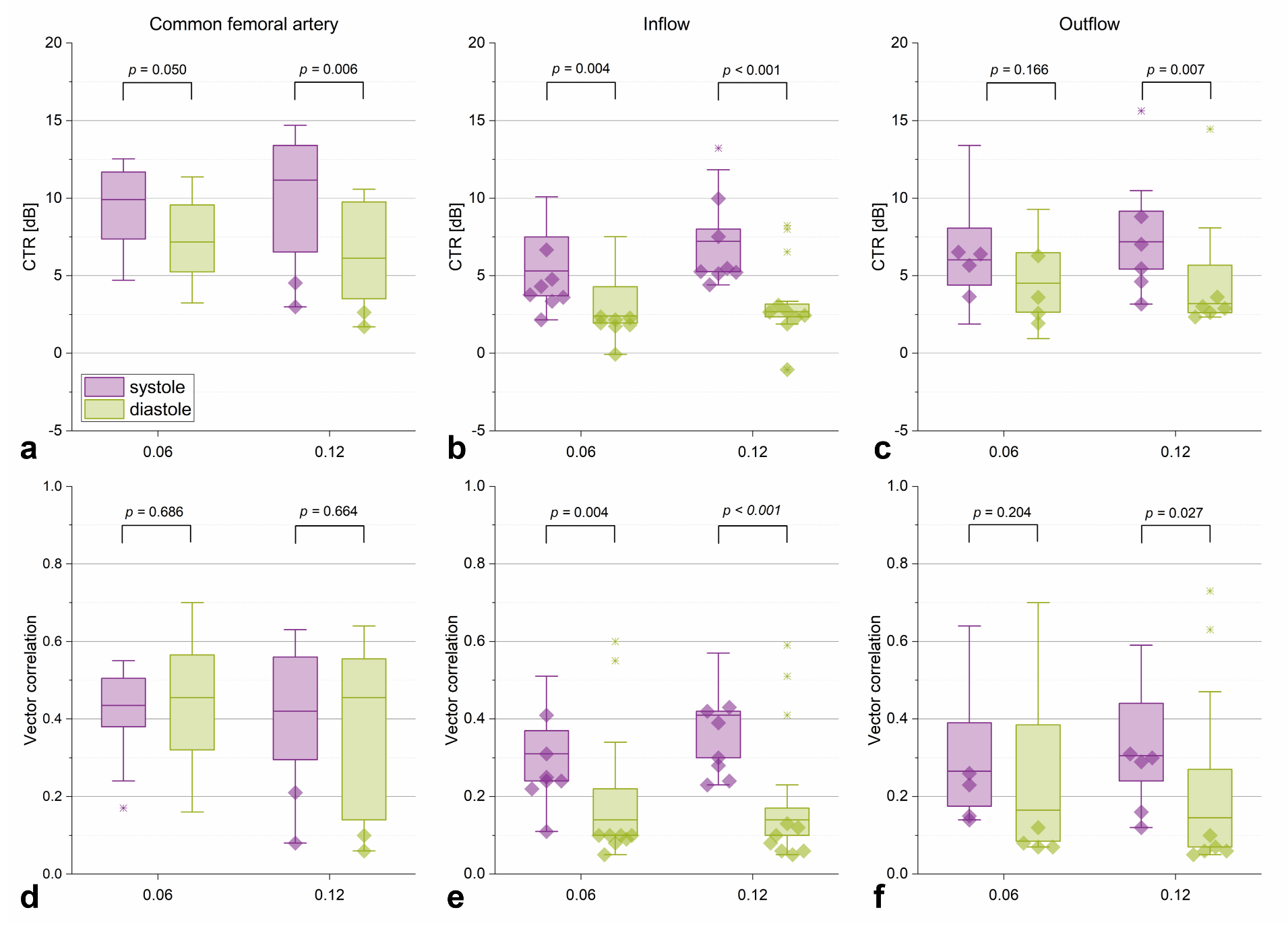


**Supplementary video legends**

**Video 1.** EchoPIV recording (2.5 s, slowed down to 16 s) of the blood flow in the common femoral artery. Vector velocity data of about 3 heart cycles is presented, as indicated by the temporal velocity profile. Distal to the stenosis (azimuth = -2 mm) recirculating blood flow is visualized. The color and size of the vectors represent the flow velocities. Vectors based on a correlation < 0.2 are given as red dots and considered to be erroneous.

**Video 2.** EchoPIV recording (2.5 s, slowed down to 16 s) of the blood flow in the common femoral artery. Vector velocity data of about 5 heart cycles is presented, as indicated by the temporal velocity profile. This patient presented with a cardiac arrhythmia. The color and size of the vectors represent the flow velocities. Vectors based on a correlation < 0.2 are given as red dots and considered to be erroneous.

**Video 3.** EchoPIV recording (2.5 s, slowed down to 16 s) of the blood flow in the common femoral artery. Vector velocity data of about 3 heart cycles is presented, as indicated by the temporal velocity profile. Velocities up to 120 cm/s are captured. The color and size of the vectors represent the flow velocities. Vectors based on a correlation < 0.2 are given as red dots and considered to be erroneous.

**Video 4.** EchoPIV recording (2.5 s, slowed down to 16 s) of the blood flow in the common femoral artery. Vector velocity data of about 2 heart cycles is presented, as indicated by the temporal velocity profile. High and disturbed (out-of-plane) blood flow velocities, caused by a stenotic lesion (azimuth = -1), cannot be adequately captured (Fig. 4a). The color and size of the vectors represent the flow velocities. Vectors based on a correlation < 0.2 are given as red dots and considered to be erroneous.

**Video 5.** EchoPIV recording (2.5 seconds, slowed down to 16 seconds) of the blood flow at the inflow of the stent in the superficial femoral artery. Vector velocity data of about 3 heart cycles is presented, as indicated by the temporal velocity profile. Only a short segment of the vessel (without the stent) is captured in the image plane (Fig. 4b). The color and size of the vectors represent the flow velocities. Vectors based on a correlation < 0.2 are given as red dots and considered to be erroneous.

**Video 6.** EchoPIV recording (2.5 s, slowed down to 16 s) of the blood flow at the inflow of the stent in the superficial femoral artery. Vector velocity data of about 3 heart cycles is presented, as indicated by the temporal velocity profile. A calcification (azimuth = from -5 to 0 mm) causes a shadow region at which the blood flow cannot be quantified throughout the entire cardiac cycle (Fig. 4c). The color and size of the vectors represent the flow velocities. Vectors based on a correlation < 0.2 are given as red dots and considered to be erroneous.

**Video 7.** EchoPIV recording (2.5 s, slowed down to 16 s) of the blood flow at the inflow of the stent in the superficial femoral artery. Vector velocity data of about 2 heart cycles is presented, as indicated by the temporal velocity profile. During diastole the microbubbles are destroyed due to the prolonged exposure time to ultrasound. Consequently, blood flow could not be visualized during this phase of the cardiac cycle (Fig. 4d). Vectors based on a correlation < 0.2 are given as red dots and considered to be erroneous.

**Video 8.** B-mode contrast recording at the inflow region of the stent in the superficial femoral artery used to monitor contrast levels and as reference for the stent location. The proximal edge of the stent can be found around 5 mm azimuth.

**Video 9.** EchoPIV recording (2.5 s, slowed down to 16 s) of the blood flow at the inflow of the stent in the superficial femoral artery. Vector velocity data of about 2 heart cycles is presented, as indicated by the temporal velocity profile. The stent was placed in the proximal superficial femoral artery and thus the femoral bifurcation is imaged. The color and size of the vectors represent the flow velocities. Vectors based on a correlation < 0.2 are given as red dots and considered to be erroneous.

**Video 10.** B-mode contrast recording at the outflow region of the stent in the superficial femoral artery used to monitor contrast levels and as reference for the stent location. The distal edge of the stent can be found around -5 mm azimuth.

**Video 11.** EchoPIV recording (2.5 s, slowed down to 16 s) of the blood flow at the outflow of the stent in the superficial femoral artery. Vector velocity data of about 3 heart cycles is presented, as indicated by the temporal velocity profile. Venous blood flow can be appreciated posterior to the superficial femoral artery. The color and size of the vectors represent the flow velocities. Vectors based on a correlation < 0.2 are given as red dots and considered to be erroneous.
